# Arteriovenous fistula in a renal allograft with gross hematuria and subsequent acute kidney injury due to urinary tract obstruction: a case report

**DOI:** 10.1186/s12882-023-03183-5

**Published:** 2023-06-05

**Authors:** Yujiro Aoki, Takeshi Kawamura, Nobuyuki Shiraga, Takashi Yonekura, Maho Maeda, Sota Kurihara, Yoshitaka Sekine, Seiichiro Shishido, Ken Sakai

**Affiliations:** 1 Department of Nephrology, Toho University Faculty of Medicine, 6-11-1 Omori-Nishi, Ota-ku, Tokyo, 143-8541 Japan; 2 Department of Diagnostic Radiology, Toho University Omori Medical Center, Tokyo, Japan; 3 Department of Urology, National Hospital Organization Takasaki General Medical Center, Takasaki, Japan; 4 Department of Urology, Gunma University Graduate School of Medicine, Maebashi, Japan

**Keywords:** Arteriovenous fistula, Kidney transplantation, Renal allograft biopsy, Acute kidney injury

## Abstract

**Background:**

Arteriovenous fistula (AVF) due to renal allograft biopsy is mechanical trauma resulting from the penetration of small arteries and veins by a core needle. Most AVFs are reported to resolve asymptomatically and spontaneously. This report presents a patient with acute kidney injury (AKI) due to urinary tract obstruction caused by a bleeding AVF in a renal allograft.

**Case presentation:**

A 22-year-old Japanese woman who underwent living-donor kidney transplantation (KT) at 3 years due to end-stage renal disease caused by focal segmental glomerulosclerosis (FSGS) presented with a renal transplant AVF (gourd-shaped; 42 × 19 × 20 mm). The AVF was unexpectedly discovered by ultrasound before a surveillance biopsy at 10 years after KT. The patient had a history of recurrent FSGS, had undergone several renal allograft biopsies after KT, and did not experience symptoms or growth of the AVF for years. Nineteen years after KT, the patient developed AKI with sudden, asymptomatic, gross hematuria and anuria. Plain computed tomography revealed a hematoma in the pelvis of the renal allograft and bladder tamponade. The AVF was successfully treated by coil embolization. Hemodialysis was performed for AKI, and graft function was gradually recovered.

**Conclusions:**

Unexpected bleeding from a renal transplant AVF may lead to transplant dysfunction. Angiographic embolization against the ruptured renal transplant AVF may prevent rebleeding and rescue the renal allograft.

## Background

Renal allograft biopsy is the best diagnostic tool to assess the degree of acute and chronic allogeneic renal dysfunction and to provide crucial diagnostic and prognostic information [1]. However, renal allograft biopsy is invasive and complicated by bleeding [2]. A blood transfusion is required in 0.3% of patients undergoing renal allograft biopsy [3]. An arteriovenous fistula (AVF) is a common complication after renal allograft biopsy [3, 4]. The incidence of renal transplant AVF is 8.3–16.7% [4–8], and approximately 70% resolve within 1–2 years, whereas 30% are persistent or symptomatic [5–9]. Embolization is an effective treatment for renal transplant AVF [5, 10], although the technical success of embolization does not always translate into clinical success due to renal dysfunction or hypertension [5, 11]. In addition, endovascular treatment may lead to complications requiring emergency surgery. Therefore, AVF treatment may not be as effective as desired, and there are controversies regarding the effectiveness of early therapeutic intervention for renal transplant AVF [11, 12].

Although severe hematuria from a renal transplant AVF after renal allograft biopsy has been reported [13, 14], sudden-onset hematuria from an asymptomatic renal transplant AVF has not been reported. This study presents a patient with sudden hemorrhage from a renal transplant AVF that resulted in acute kidney injury (AKI). The patient was treated by a multidisciplinary team, and the allograft was saved by embolization.

## Case presentation

**Fig. 1:**
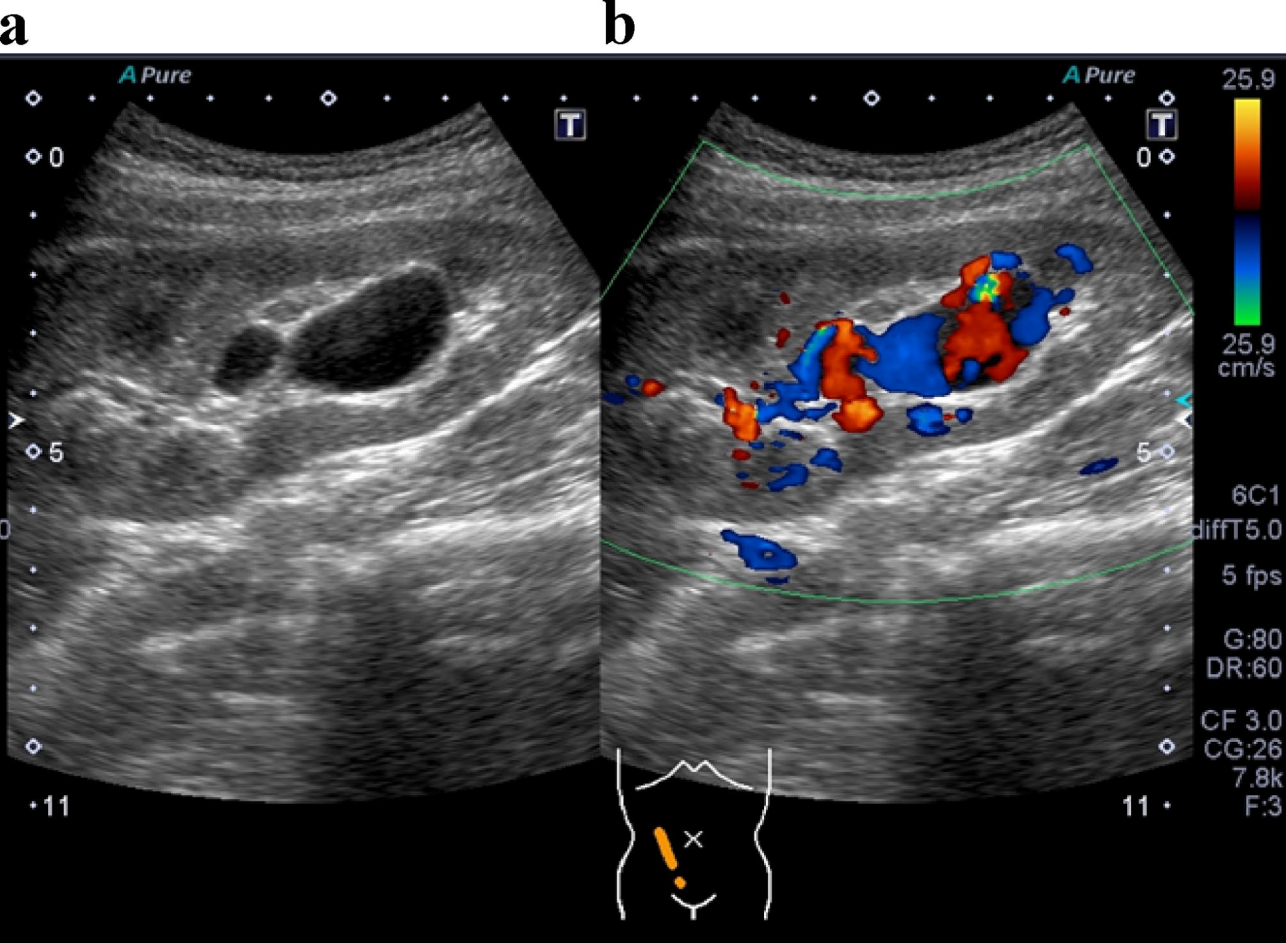
Initial ultrasonography image of the renal transplant AVF. **(a)** A renal transplant AVF 10 years after kidney transplantation. **(b)** A color Doppler ultrasonography image shows an aneurysmal dilatation (42 × 19 × 20 mm) in the lower pole of the allograft AVF: arteriovenous fistula

A 22-year-old female kidney transplant recipient was admitted to our facility for sudden gross hematuria and AKI. The patient developed nephrotic syndrome at 18 months and was diagnosed with focal segmental glomerulosclerosis (FSGS). Due to rapid deterioration of her renal function, the patient underwent peritoneal dialysis for end-stage kidney disease 5 months after symptom onset. At 39 months, the patient underwent an ABO-compatible living-donor kidney transplantation (KT). However, the patient developed heavy proteinuria and hypoalbuminemia within 24 h of the KT and experienced recurrent FSGS. A non-randomized, new treatment protocol comprising steroid pulse and high-dose cyclosporine A-based quadruple immunosuppression was administered [15]. The patient achieved complete remission 14 months after treatment for recurrence. Typically, kidney biopsies are performed at the time of recurrence, and screening biopsies are performed 3 months, 1 year, and 2 years after KT. Two core biopsies are typically obtained using a spring-loaded 16-gauge biopsy gun under ultrasound guidance. Thereafter, surveillance biopsies are performed at 3, 5, and 10 years after KT. At the time of FSGS recurrence in this patient, a renal allograft biopsy revealed a subcapsular hematoma. The patient was managed conservatively without hematoma growth or hematuria. Subsequent follow-up examinations showed no hematuria or AVF. The patient was admitted for a surveillance biopsy 10 years after KT, and color-coded Doppler ultrasonography detected an AVF as a mosaic signal in the lower pole of the allograft (Fig. 1a). The gourd-shaped AVF was 42 × 19 × 20 mm, with a continuous artery running from the renal portal with pulsatile blood flow signals (Fig. 1b). Although the AVF was considered massive, the patient had no symptoms associated with renal transplant AVF, including hematuria, abdominal pain, hypertension, or renal impairment. Interventional radiology was contraindicated owing to the risk of loss of renal transplant function due to embolization of the renal transplant AVF. Further, there were concerns regarding complications of vascular therapy. A subsequent renal allograft biopsy was not performed, and the patient was followed up for growth and symptomatology of the AVF by ultrasound examinations. Ultrasonography confirmed after 4 and 7 years that the AVF had not increased in size (42 × 22 mm and 40 × 25 mm, respectively).

**Fig. 2:**
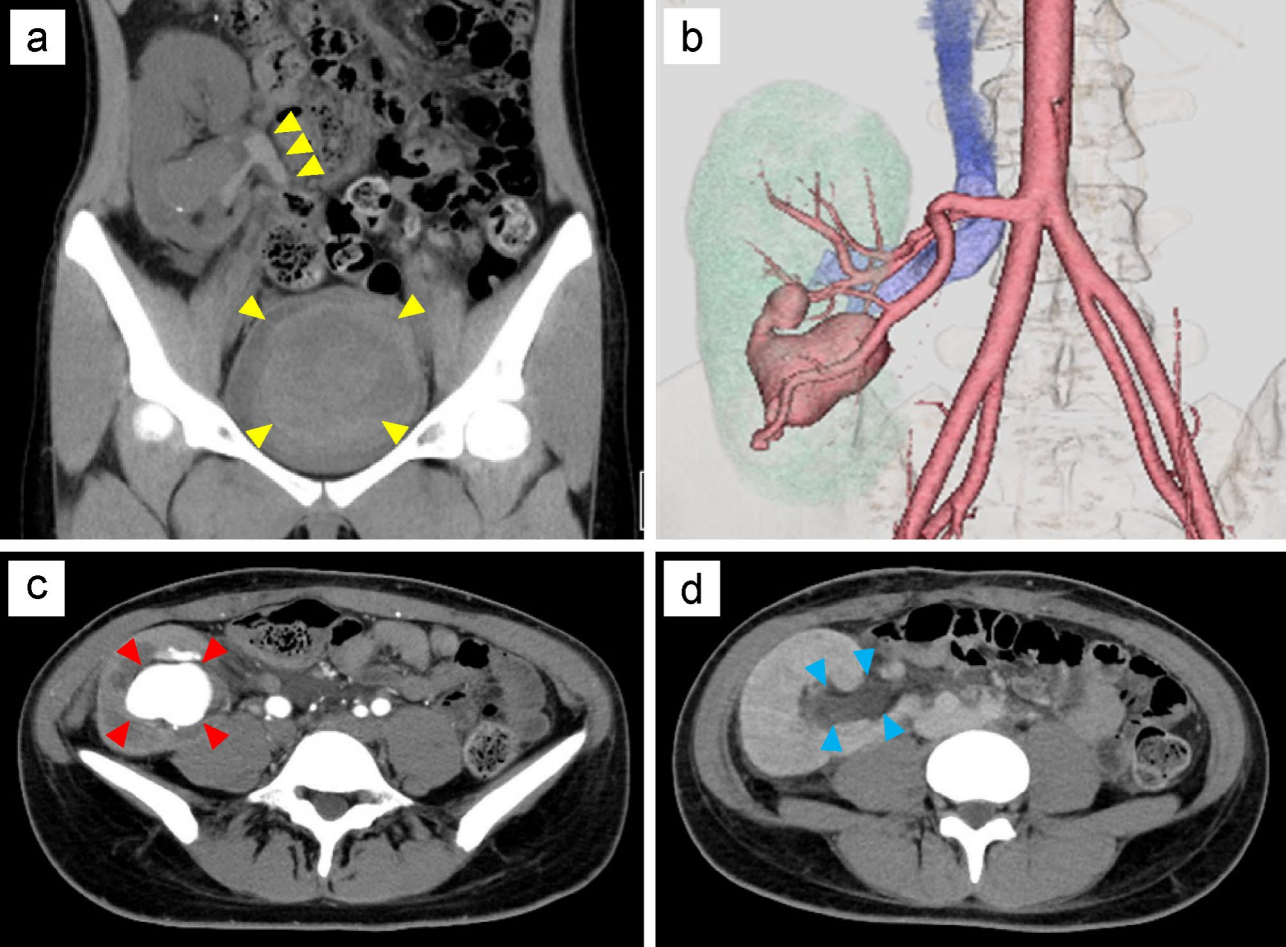
CT image after the onset of symptoms. **(a)** A plain CT image shows a hematoma in the transplanted renal ureter and bladder tamponade (yellow arrowhead). **(b)** A three-dimensional CT image shows a large, gourd-shaped cystic structure and renal transplant AVF inflow from the renal artery. c, d. Dynamic contrast-enhanced CT shows the morphologic features of an enlarged renal transplant AVF (red arrowhead) and dilated renal vein (blue arrowhead) AVF, arteriovenous fistula; CT, computed tomography

**Fig. 3:**
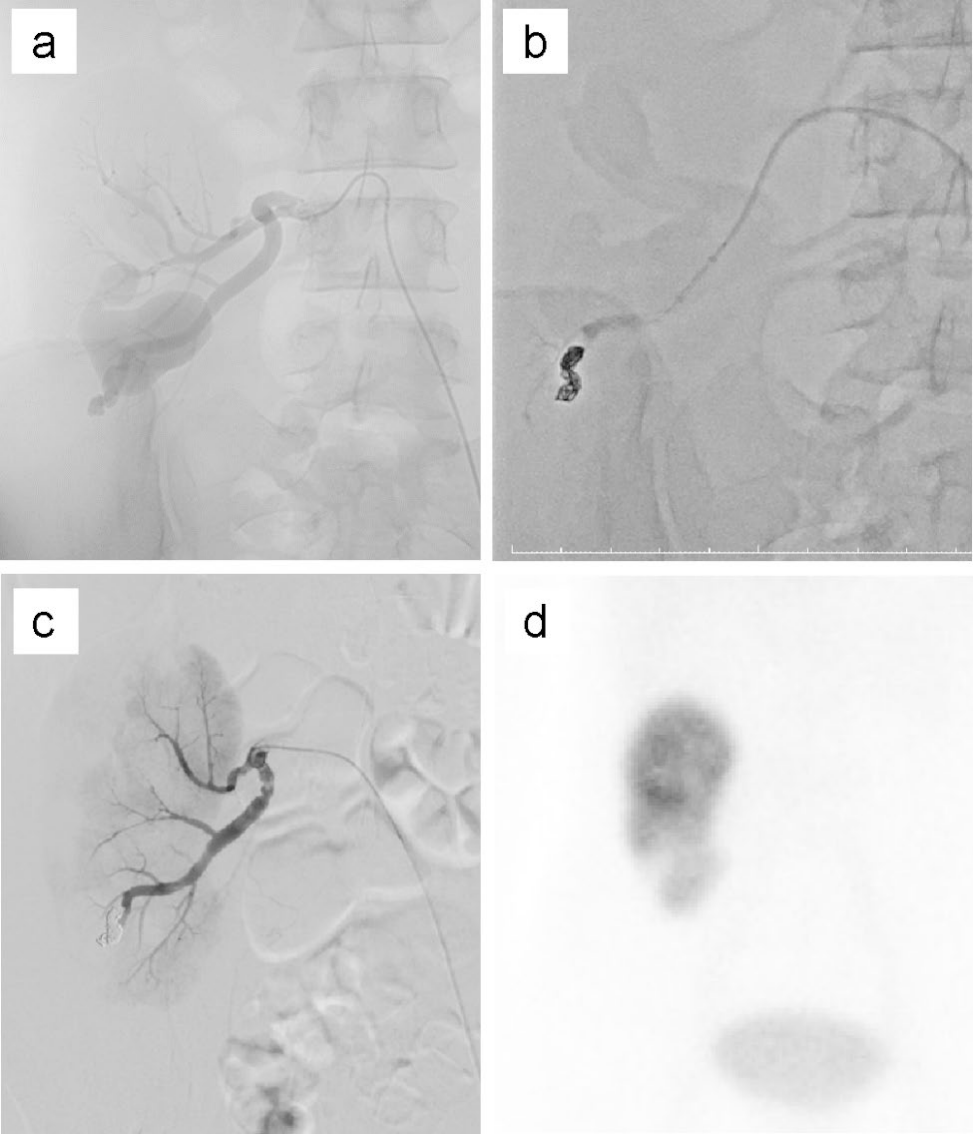
Embolization therapy for a renal transplant AVF and the post-embolization DMSA image. **(a)** A selective renal transplant angiogram shows an AVF supplied by a lobular artery in the lower pole of the allograft. **(b)** Detachable platinum coils are placed in the renal artery branch feeding the AVF. **(c)** After angiographic embolization, the AVF is not detected because it is completely embolized. **(d)** DMSA scintigraphy shows a defect in the lower-third of the allograft AVF, arteriovenous fistula; DMSA, dimercaptosuccinic acid

**Fig. 4:**
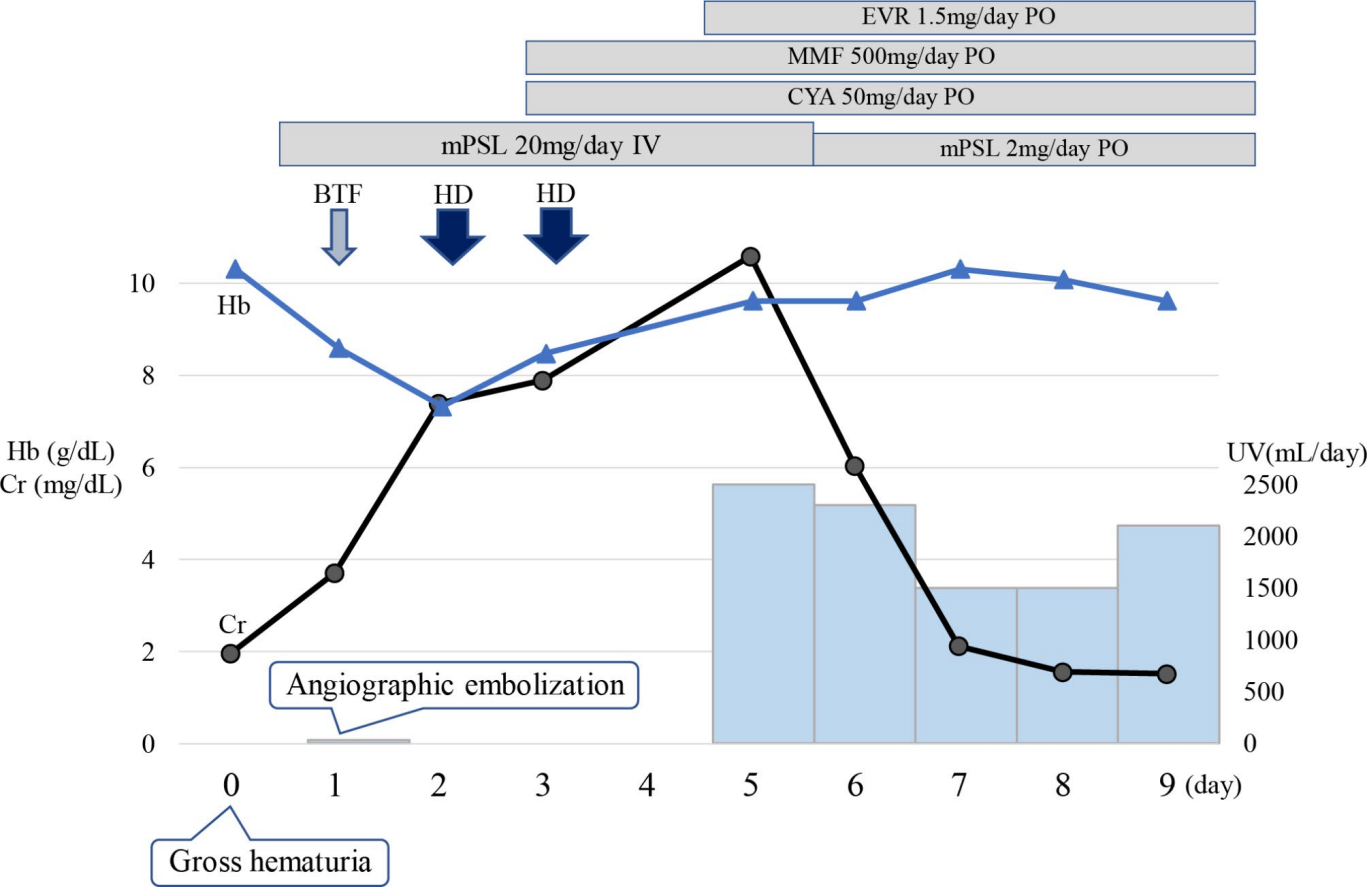
Clinical course after onset. After angiographic embolization, hemodialysis was performed and renal transplant function was improved

Nineteen years after KT, the patient presented with sudden asymptomatic gross hematuria in the evening. The patient reported feeling nauseated and experiencing dysuria; she was referred to a general hospital. Plain computed tomography (CT) showed hematoma in the renal pelvis of the allograft and bladder tamponade (Fig. 2a), and the patient was transported to our hospital. Upon admission to our hospital, her blood pressure was 170/101 mmHg, pulse 84 bpm, and body temperature 38.3℃. On physical examination, the patient had graft tenderness and persistent anuria. The patient was conscious with clear awareness. Her white blood cell count was 7,400/µL, red blood cell count 3.17 × 10^4^/µL, hemoglobin 8.2 g/dL, hematocrit 27.8%, platelet count 20.3 × 10^4^/µL, blood urea nitrogen 33 mg/dL, creatinine 3.71 mg/dL, estimated glomerular filtration rate 14.1 mL/min/1.73 m^2^, and C-reactive protein 0.1 mg/dL. Although her vital signs were stable, the anuria was prolonged, and blood tests performed 7 h after the patient’s admission showed decreased hemoglobin (7.5 g/dL) and renal function (creatinine, 4.92 mg/dL). A 35-mm AVF was observed on ultrasound. Dynamic contrast-enhanced and three-dimensional CT scans (Fig. 2b) delineated a gourd-shaped mass in the allograft kidney (Fig. 2c) and marked dilatation of the left renal vein (Fig. 2d). The patient was believed to have developed AKI due to urinary tract obstruction caused by bleeding or hematoma from the renal transplant AVF. Therefore, she underwent emergency angiographic embolization. Transcatheter embolization of the AVF was performed by a radiologist using detachable coils. The Excelsior® 1018 Microcatheter (Stryker, Fremont, CA, USA) was introduced in the AVF, and embolization was performed with Target XXL® 360 (5 mm×20 cm; Stryker, Fremont, CA, USA) coils and Target XL® 360 Soft (4 mm×12 cm, 3 mm×9 cm; Stryker, Fremont, CA, USA) coils. Renal allograft angiography after embolization showed a diminished AVF. The AVF was successfully blocked, with renal blood flow conserved (Fig. 3). The patient remained anuric and underwent two cycles of hemodialysis. She had urinary outflow, and her serum creatinine level decreased after postoperative day 5. On postoperative day 8, dimercaptosuccinic acid scintigraphy revealed a defect in the lower-third of the allograft (Fig. 3d). The patient’s general condition and graft function subsequently improved (creatinine, 1.57 mg/dL). Ultimately, the patient was discharged on postoperative day 18 without adverse events. The clinical course is shown in Fig. 4.

## Discussion and conclusions

This case report presents a patient with AKI due to urinary tract obstruction caused by bleeding from a renal transplant AVF, which was successfully treated by embolization. Notably, the bleeding occurred 9 years after a renal allograft biopsy, with no history of microscopic hematuria, stable blood pressure and renal function, and no changes in the renal transplant AVF until the patient’s presentation.

Although renal allograft biopsy is widely considered the best method for evaluating a transplanted kidney, one of the vascular complications associated with the procedure is the formation of an AVF, which occurs in 8.3–16.7% of patients undergoing renal allograft biopsy [4–8]. In most patients, the AVF resolves spontaneously, although they remain symptomatic in 30% of patients [6]. Hematuria is the most common symptom of renal transplant AVF [4–6], causing renal dysfunction, hypertension, and hemorrhagic shock due to bleeding from the AVF [13, 14]. AVF often occurs early to 1 year after renal allograft biopsy [4, 5, 9, 13, 14], and a history of multiple renal allograft biopsies has been reported as a high-risk factor for AVF development [16, 17].

The patient in this report underwent living-donor KT at 3 years using an intraperitoneal approach, followed by seven surveillance biopsies. As the intraperitoneal approach was used, the peritoneum and intestinal tract covered the anterior surface of the transplanted kidney, which may have resulted in a biopsy of a localized area of the lower pole of the allograft. A renal transplant AVF was detected during surveillance biopsy 10 years after KT in this patient, although approximately 9 years had passed before the onset of gross hematuria. The patient was not prescribed antihypertensive medication, her blood pressure was maintained at near 120/70 mmHg, and her serum creatinine level was stable at approximately 1.5 mg/dL. Furthermore, no microscopic hematuria was observed during the outpatient course. During a routine visit 20 days before the disease onset, the patient’s blood pressure was elevated at 147/107 mmHg, although no microscopic hematuria, worsening renal function, or fluid retention was observed. Renal AVFs lead to abnormal venous and arterial traffic. Further, the reduction in venous vascular resistance reduces blood flow through the renal parenchyma, causing renal ischemia, activation of the renin-angiotensin system, hypertension, and renal failure [18]. In addition, the vascular steal phenomenon caused by AVF increases venous return and may play a role in high-output heart failure [19]. Therefore, the elevated blood pressure before the disease onset in this patient may have been a predictor of symptomatic changes in renal transplant AVF.

Voiculescu et al. [13] reported a case of renal transplant AVF hemorrhage with gross hematuria similar to the present case. In this case, a kidney transplant biopsy was performed due to suspected rejection after KT, and a subsequent ultrasound scan revealed an AVF. During follow-up, the patient had multiple urinary tract infections and was treated in the hospital. Eight months later, she suddenly developed massive hematuria, bladder tamponade, hemorrhagic shock, and urinary tract sepsis. The bleeding from the renal transplant AVF was stopped by embolization. The authors noted that multiple urinary tract infections under immunosuppressive therapy after KT may have caused the AVF tissue to become fragile and hemorrhagic. In our experience, there was no history of urinary tract infection after KT. There was also no concurrent urinary tract infection at the disease onset, making it unlikely that the AVF tissue was fragile due to infection. Furthermore, 9 years had passed from the last kidney transplant biopsy to the disease onset, and the renal transplant AVF had not increased. However, the patient had mildly elevated blood pressure at the outpatient visit, suggesting that the transient increase in blood pressure may have caused the renal transplant AVF to rupture.

Few studies have reported the efficacy and safety of embolization for treating renal transplant AVF [10, 20]. In contrast, the management of asymptomatic renal transplant AVF is controversial, with proposed treatment methods not always improving renal function [12, 20]. Furthermore, endovascular therapy is associated with complications that may require emergency surgery [11]. Contrast media use in interventional radiology may lead to worse renal function and unexpected enlargement of the renal infarction after embolization. However, embolization is recommended for symptomatic renal transplant AVF due to the associated risk of bleeding, renal ischemia, and heart failure [20]. We monitored the patient for a large asymptomatic renal transplant AVF. In addition, no symptomatic changes were noted during the treatment course, and it was difficult to predict the rupture of the renal transplant AVF. After the rupture of the renal transplant AVF, anemia and renal function worsened, but there was no persistent bleeding. However, given the risk of rebleeding, angiographic embolization was performed on the renal transplant AVF. As a result, hemodialysis for AKI could be performed safely, and the renal allograft was salvaged.

In conclusion, unexpected bleeding from the renal transplant AVF leads to renal dysfunction and reduced quality of life. Angiographic embolization against the rupture of the renal transplant AVF may prevent rebleeding and rescue the renal allograft.

## Data Availability

The datasets used and/or analyzed during the current study are available from the corresponding author on reasonable request.

## Abbreviations

AKI: acute kidney injury
AVF: arteriovenous fistula
CT: computed tomography
FSGS: focal segmental glomerulosclerosis
KT: kidney transplantation
